# Nondestructive Detection of Moldy Pear Core for Fruit Quality Control Using Vis/NIR Spectroscopy and Enhanced Image Encoding via Deep Learning

**DOI:** 10.3390/foods15101756

**Published:** 2026-05-15

**Authors:** Congkai Liu, Kang Zhao, Yunhao Zhang, Wenbo Fu, Shuhui Bi, Ye Song

**Affiliations:** 1 School of Electrical Engineering, University of Jinan, Jinan 250022, China; a2661853870@163.com (C.L.); f17353619403@163.com (Y.Z.); 15550055096@163.com (W.F.); cse_bish@ujn.edu.cn (S.B.); 2 Jinan Fruit Research Institute, All-China Federation of Supply and Marketing Cooperatives, Jinan 250220, China; songye_sy@163.com

**Keywords:** moldy pear core, visible near-infrared spectroscopy, deep learning, nondestructive detection

## Abstract

Moldy pear core constitutes a severe internal defect that compromises fruit quality. This study proposes a nondestructive detection method for Korla pear moldy core using Vis/NIR spectral signals, aimed at supporting post-harvest quality control and automated industrial sorting. We collected spectral signals from pears and quantified the moldy pear core area to classify samples into healthy (S = 0%), slightly moldy (0 < S ≤ 10%), and severely moldy (S > 10%) categories. We constructed a three-tier comparative framework to evaluate the progression from conventional machine learning to advanced deep learning: traditional methods using univariate selection (US) and random forest (RF) for feature extraction followed by support vector machine (SVM) classification; 1D-ResNet for direct processing of spectral signals; and two-dimensional approaches transforming signals into improved gramian angular field (IGAF) or Laplacian pyramid Markov transition field (LPMTF) images processed through deep belief network (DBN), MobileNetv3, and Vision Transformer (ViT). The LPMTF-ViT combination delivered the best performance with 98.98% test accuracy and 94.44% external validation accuracy, significantly exceeding traditional approaches and 1D-ResNet. This innovative approach delivers effective technical support for early-stage, nondestructive detection of internal fruit defects. It also establishes a scalable foundation for automated industrial inspection systems, potentially reducing post-harvest losses while ensuring premium quality control in modern fruit supply chains.

## 1. Introduction

Pear is one of the world’s most economically important fruit crops. Global production exceeds 16 million tons annually [1]. China dominates this sector, accounting for nearly 70% of worldwide output. For example, high-quality varieties of Korla pears can achieve very high market prices [2]. However, the pear industry faces serious challenges, particularly from moldy pear core. This physiological disorder, caused by infection with Alternaria alternata, affects the core tissues of the fruit [3]. The disease progressively reduces internal fruit quality while remaining undetectable through external visual inspection during its early stages [4]. Consequently, it results in economic losses of 15–30% in severely affected regions. Additionally, infected fruits pose health risks to consumers, as fungal pathogens can produce mycotoxins such as patulin (Penicillium expansum) and Alternaria toxins (Alternaria alternata), which are associated with gastrointestinal, immunotoxic, and potentially carcinogenic effects [5].

Post-harvest fungal pathogens cause substantial economic losses in the global fruit industry. Botrytis cinerea and Penicillium expansum, the primary causative agents of gray mold and blue mold, result in significant rot losses in grape berries and jujube fruit during storage and transportation [6]. Similarly, various post-harvest pathogens are reported to cause considerable losses at different storage stages, accounting for nearly 50% of wastage in citrus fruits [7]. To mitigate these risks, several post-harvest preservation strategies have been developed, including refrigeration, ionizing radiation, edible coating technologies, chemical treatments, fungicide application, ultrasound technology, ozone treatments, pulsed electric fields, and cold plasma technology [8].

Furthermore, traditional methods for assessing fruit quality, such as manual visual sorting, laboratory chemical analysis, and professional equipment testing, are considered reliable. However, they are inherently destructive, labor-intensive and time-consuming, making them unsuitable for large-scale industrial application [9]. Nevertheless, all the above approaches are primarily preventive in nature and cannot remediate fruits that are already infected. Therefore, early and nondestructive detection of internal infections remains a critical unmet need in modern fruit supply chains.

In response to these challenges, nondestructive detection technologies have emerged as promising solutions for assessing fruit quality. Hyperspectral imaging has shown high accuracy in detecting internal defects in apples and oranges [10]. Moreover, X-ray computed tomography effectively identifies internal cavities and density variations in stone fruits [11,12]. Additionally, magnetic resonance imaging demonstrates potential for non-invasive assessment of water distribution and tissue integrity in pears [13,14]. THz inspection technology also plays an important role in the detection of pine nuts and sunflower seeds [15,16].

Among nondestructive detection techniques, Vis/NIR spectroscopy has attracted considerable attention for fruit quality assessment due to its advantages of low cost, rapid acquisition, non-contact measurement, and suitability for online industrial applications. Moreover, it enables simultaneous acquisition of physical and chemical information from intact fruits without damage, making it well-suited for large-scale post-harvest quality inspection. Consequently, Vis/NIR spectroscopy has been widely adopted for detecting various internal quality attributes in fruits, including soluble solid content, acidity, and internal defects.

However, most studies focus on other fruit species or defect types, showing a research gap regarding moldy pear core detection in pears through systematic comparative approaches.

Deep learning has achieved remarkable success in image recognition tasks in recent years, leading to its adoption across multiple disciplines [17,18]. A key advantage of deep learning is its ability to extract complex spatial features from two-dimensional representations. This significantly improves detection accuracy compared to traditional one-dimensional signal processing methods [19]. This approach is particularly effective when spectroscopic data are transformed into image-like formats that preserve both local and global signal characteristics. For instance, deep convolutional generative adversarial networks have been applied to near-infrared hyperspectral imaging for predicting the oil content of individual maize kernels [20], and multi-level dynamic feature extraction strategies have further enhanced the quality prediction performance of deep learning models in near-infrared spectroscopy analysis [21].

Despite these advancements, conventional encoding methods often suffer from temporal characteristic overlaps or overemphasize local details while losing global spectral patterns [22]. Recent improvements, such as wavelet-enhanced Recurrence Plot (RP) and optimized GAF, have shown promise in other applications [23,24]. While both GAF and MTF have been applied to encode spectral time series, systematic comparisons of their performance specifically for fruit quality detection remain limited. Furthermore, studies integrating such encoding comparisons within a unified framework contrasting 1D-CNN and deep Learning model architectures are scarce. Consequently, which combination of encoding strategy and network architecture yields optimal performance for specific fruit spectral datasets remains insufficiently understood. The lack of standardized, head-to-head evaluations under identical experimental conditions hinders evidence-based selection of optimal detection strategies.

This study explores the feasibility of integrating Vis/NIR spectroscopy with improved image encoding techniques for nondestructive identification of moldy pear core. The primary objective is to systematically compare three distinct analytical paradigms for processing spectral data: conventional machine learning, 1D deep learning, and 2D image encoding-based deep learning. This comparison aims to identify the optimal transformation that preserves essential spectral characteristics. We establish a comprehensive three-tier comparative framework: (1) traditional machine learning using characteristic wavelength extraction with SVM as a baseline [25]; (2) a 1D-CNN based on the 1D-ResNet architecture applied directly to spectral data; and (3) Deep Learning model utilizing IGAF and LPMTF transformations, combined with three feature extractors (DBN, MobileNetv3, and ViT) for classification. This systematic comparison aims to provide the best detection strategy and contribute to the development of nondestructive fruit quality assessment technology.

## 2. Materials and Methods

### 2.1. Sample Acquisition and Experimental Grouping

Korla pear specimens were acquired in November 2024 from Hualian Fruit Wholesale Market in Jinan City, Shandong Province. We selected 1300 fruits with intact surfaces and without external injuries. These were subsequently delivered to the laboratory through cold-chain transportation, assigned individual codes, and maintained in a climate-controlled chamber at 25 °C for 24 h to stabilize their internal constituents. Among them, 650 pear samples were immediately subjected to spectral signal detection and moldy pear core area calculation as healthy samples, while the remaining 650 pear samples were incubated at room temperature (25 ± 1 °C) for three days before undergoing detection and moldy pear core area calculation.

### 2.2. Response Signal Acquisition of Vis/NIR Spectra of Korla Pear

To achieve nondestructive detection of internal moldy pear cores in pear fruits, a Vis/NIR spectroscopy-based acquisition platform was constructed. The system utilized two 41850SP-type tungsten halogen lamps (Osram, Munich, Germany) mounted at the top of a dark chamber as the excitation source. These lamps emitted strong, continuous radiation across the visible to near-infrared region, particularly at wavelengths above 700 nm, providing sufficient penetration depth for biological tissues such as Korla pears. The thin skin and relatively translucent flesh of this cultivar facilitate deep light penetration toward the core, thereby providing a physical basis for the feasibility of non-invasive moldy core detection. Prior to spectral acquisition, the light sources were preheated for 60 min to ensure output stability.

The sample stage, positioned below the illumination sources, was height-adjustable and featured a central through-hole, allowing an optical fiber probe to effectively collect transmitted light passing through the fruit. A QE Pro miniature spectrometer (Ocean Optics, Dunedin, FL, USA) with a signal-to-noise ratio of 1000:1 was employed to disperse and detect the transmitted light across the target wavelength bands. The detector maintained highly stable temperature regulation, ensuring optimal performance during long-duration measurement sessions. The digitized spectral signals were subsequently transmitted to a computer for recording and storage.

Spectral signals were acquired using an integration time of 300 ms, covering the wavelength range of 350–1150 nm. Considering that moldy pear core tissues are often irregularly distributed within the fruit and may deviate from the central region, three transmission spectra were collected for each Korla pear by rotating the fruit sequentially around its stem–calyx axis. This multi-point measurement strategy reduced systematic deviations caused by single-measurement orientation and tissue heterogeneity. The arithmetic mean of these three spectra was calculated as the final spectral signature for each sample.

**Figure 1:**
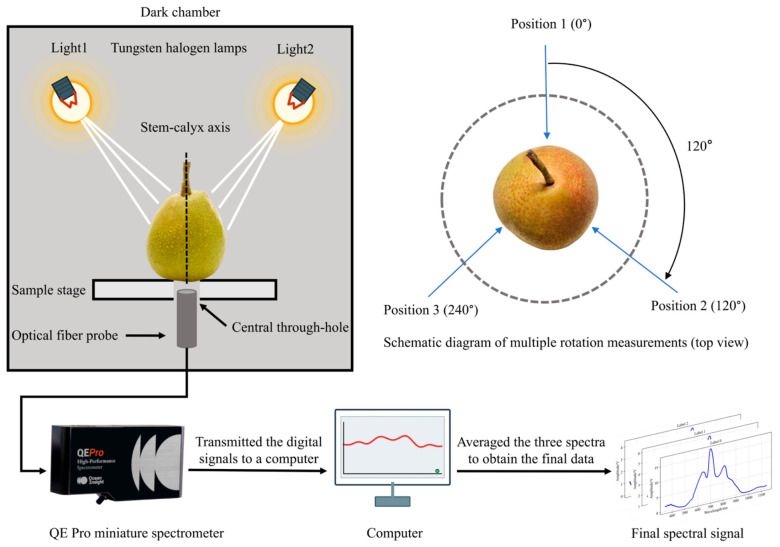
Spectral signal acquisition process.

Following data collection, 52 samples exhibiting excessively large signal deviations or abnormal values were manually identified and excluded as outliers, yielding 1248 valid samples for subsequent model training. The spectral signal acquisition process is illustrated in Figure 1.

### 2.3. Quantification and Grading of Moldy Pear Core

**Figure 2:**
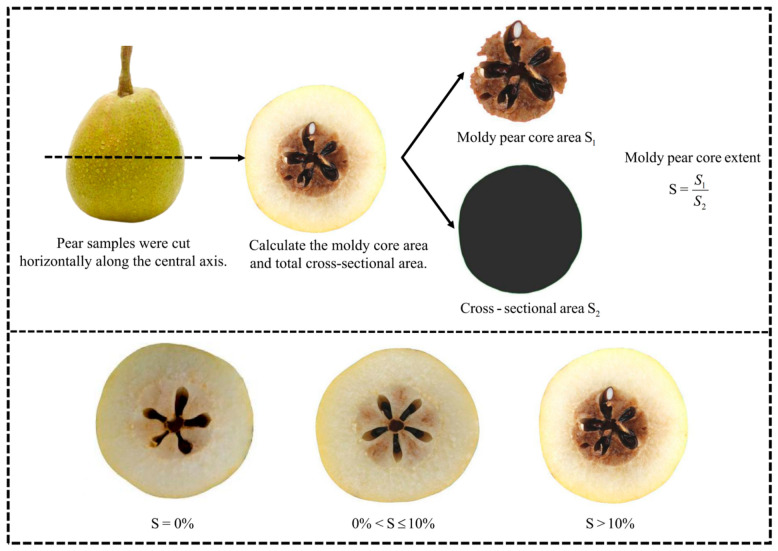
Quantification method of moldy pear core extent in pears.

Following the acquisition of Vis/NIR spectroscopy signals, each sample was horizontally cut along its central axis. The cross-section was photographed with a digital camera to quantify the extent of moldy pear core. The computational procedure is depicted in Figure 2. RGB tri-channel components of the pear images were extracted. Subsequently, Canny edge detection was used to trace cross-sectional contours. B-channel images were segmented using iterative thresholding to enhance visualization of moldy pear core regions. An 8-connectivity component labeling algorithm was utilized to identify and extract moldy pear core areas from the segmented images. Finally, the percentage of moldy pear core area was calculated, where $S_{1}$ represents the area of the moldy region, and $S_{2}$ represents the total cross-sectional area of the pear. The degree of deterioration for each Korla pear was calculated by the ratio $S=\frac{S_{1}}{S_{2}}\times 100\%$. For the purposes of this study, pears with S = 0% were classified as healthy, those with 0% < S ≤ 10% were categorized as slightly moldy, and specimens with S > 10% were classified as severely moldy.

### 2.4. Extraction Method of Characteristic Wavelength

Feature wavelength extraction is an effective spectral data analysis technique. It can identify and select the most discriminative wavelengths for classification tasks by leveraging the informative patterns within spectral signals. However, standard spectral analysis suffers from redundancy due to highly correlated adjacent wavelengths. This study employed feature wavelength extraction to resolve the original spectral signal into a series of discriminative and non-discriminative wavelength components. These components were subsequently mapped onto respective feature wavelength sets using two methods: univariate selection and random forest importance assessment.

**Figure 3:**
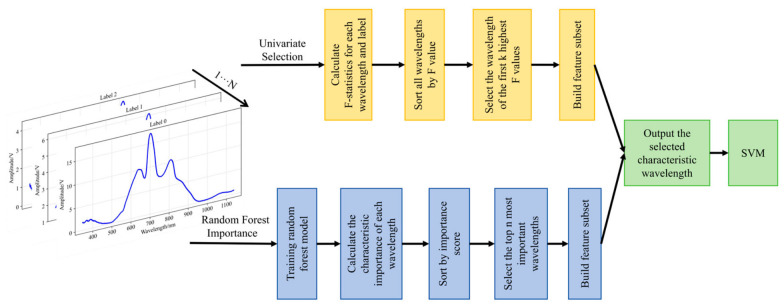
Construction of wavelength feature extractor.

The univariate feature selection method evaluates the discriminative power of each wavelength using the F-statistic. It then selects the K most statistically significant wavelengths as the feature subset. This method has high efficiency and can quickly identify wavelengths with strong discriminative power. The random forest importance assessment method leverages the advantages of ensemble learning by constructing multiple decision trees to evaluate the comprehensive importance of features. This technique captures nonlinear relationships and interactions between wavelengths, providing a robust feature selection basis. Subsequently, the selected feature wavelengths are used to construct a reduced-dimensional feature representation for classification. Finally, these features are classified using the traditional machine learning algorithm SVM. The details are shown in Figure 3.

### 2.5. Conversion of Vis/NIR Spectral Signals into Feature Images

#### 2.5.1. Improved Gramian Angular Field

GAF serves as a feature mapping strategy that converts 1D time-series signal data into 2D images [26], encompassing both GASF and the GADF. The computational procedures for GADF and GASF are demonstrated in Formulas (1) and (2). Here, I denotes the unit row vector [1, 1, …, 1], and ${\tilde{X}}^{T}$ denotes the transpose matrix of $\tilde{X}$. Given that both GASF and the GADF exhibit symmetrical characteristics, this study employed a modified GAF two-dimensional representation approach. This approach preserves the upper triangular components from GADF along with the lower triangular components from GASF, designated as IGAF, as depicted in Formula (3).

(1) $$GADF=\left[\sin(\varphi_{i}-\varphi_{j})\right]=\sin\left(\varphi_{i}(t)-\varphi_{j}(t)\right)={\sqrt{I-{\tilde{X}}^{2}}}^{T}\cdot \tilde{X}-{\tilde{X}}^{T}\cdot \sqrt{I-{\tilde{X}}^{2}}$$

(2) $$GASF=\left[\cos(\varphi_{i}+\varphi_{j})\right]=\cos\left(\varphi_{i}(t)+\varphi_{j}(t)\right)={\tilde{x}}^{T}\cdot \tilde{x}-{\sqrt{I-{\tilde{x}}^{2}}}^{T}\cdot \sqrt{I-{\tilde{x}}^{2}}$$

(3) $$\begin{array}{c} IGAF=GADF⊕GASF=\left\{\begin{array}{cc} \sin\left(\varphi_{i}-\varphi_{j}\right) & \;\;\;i\leq j\\ \cos\left(\varphi_{i}+\varphi_{j}\right) & i>j \end{array}\right. \end{array}$$

**Figure 4:**
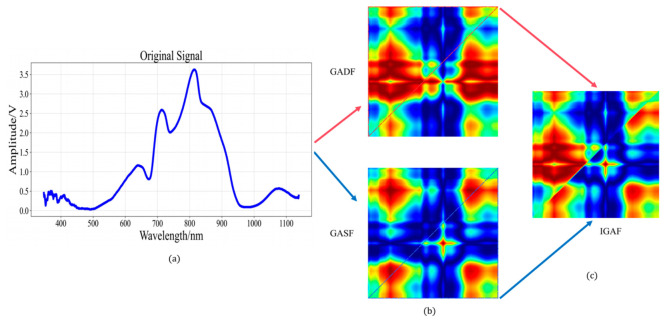
IGAF architecture schematic: (**a**) original signal, (**b**) GADF and GASF, (**c**) IGAF.

IGAF combines the topological feature representations from GADF and GASF. Through this fusion, it becomes possible to simultaneously capture both the similarities and differences within the time series, thereby enhancing discriminative capability and improving the expressiveness of the resulting images. The schematic illustration appears in Figure 4.

#### 2.5.2. Laplacian Pyramid Markov Transition Field

LPMTF constitutes an approach for transforming 1D time series into multi-scale 2D image representations. It integrates Markov transition probability matrices with Laplacian pyramid decomposition to effectively characterize the transition relationships and hierarchical features of data points within Vis/NIR spectral signals [27,28].

The Vis/NIR spectral sequence of pears with length N was preprocessed by the piecewise aggregation approximation method. In this study, the window_size is set to 5 for segmented average, which not only effectively reduces the computational complexity, but also retains the main characteristics of the signal. Discretization based on global quantiles was used, dividing the normalized time series into q equal-frequency quantile intervals. The quantile number q = 12 and window_size = 5 were determined through preliminary experiments, balancing computational efficiency with retention of discriminative spectral features. A smaller q resulted in loss of subtle spectral variations and made it difficult for the model to capture enough temporal features, while a larger window_size excessively smoothed critical peak features. The uniform distribution principle ensured consistent data volume across intervals, resulting in more uniform and stable transition probabilities in the state transition matrix across different quantile intervals.

**Figure 5:**
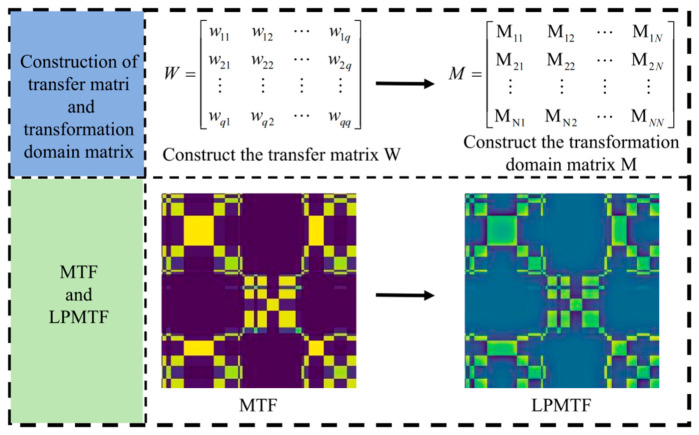
LPMTF framework schematic.

A q × q Markov transition probability matrix W is constructed based on the state transition relationship between adjacent time points, where W[i, j] represents the probability of transitioning from state i to state j. After normalization, the MTF matrix is expanded into an N × N Markov transition field M, where $M[i,j]=M[S_{i},S_{j}]$, and $s_{i}$ and $s_{j}$ are the discretized states at times $t_{i}$ and $t_{j}$, respectively. A three-layer Laplacian pyramid decomposition is performed on the normalized MTF image. First, a Gaussian pyramid is constructed through continuous down-sampling, and then the difference between adjacent layers is calculated to generate a Laplacian pyramid, resulting in multi-scale layers $L_{0}$, $L_{1}$, $L_{2}$, and $G_{3}$, which capture signal features in different frequency ranges. A weighted fusion strategy is used to integrate the information from each layer. After unifying the size of all layers, the final LPMTF image is generated, and the output result remains in the [0, 1] interval. The overall process is shown in Figure 5.

### 2.6. Deep Feature Extractor Architecture

#### 2.6.1. 1D Feature Extractor Architecture

**Figure 6:**
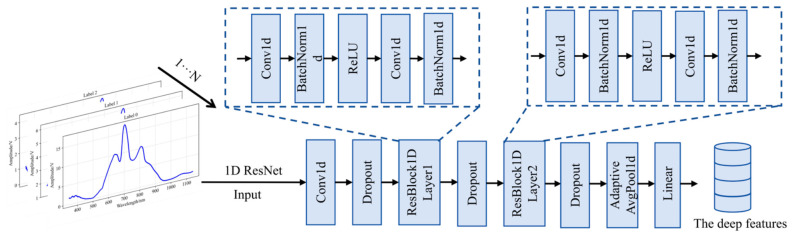
Structural diagram of 1D deep feature extractor.

To extract deep-level features from the Vis/NIR spectroscopy signals of pear cores, this study designed a residual network-based 1D feature extractor, 1D-ResNet, which directly extracts deep features of pear cores with varying degrees of mold contamination from raw 1D Vis/NIR spectral signals. The construction procedure for the 1D feature extractor is depicted in Figure 6. 1D-ResNet employs residual connection mechanisms and 1D convolution operations to capture temporal information and local patterns within the signals. Through the introduction of the ResBlock1D residual block architecture, the network effectively addresses the vanishing gradient problem encountered during deep network training, thereby enhancing the stability of feature extraction. Unlike conventional fully connected networks, 1D-ResNet uses 1D convolution kernels. This design helps maintain the temporal characteristics of signals while extracting features progressively from shallow to deep levels. Concurrently, the implementation of batch normalization and dropout techniques enhances the model’s generalization capability, reduces overfitting risks, and improves the efficacy of deep feature extraction.

#### 2.6.2. Design of 2D Feature Extractor

**Figure 7:**
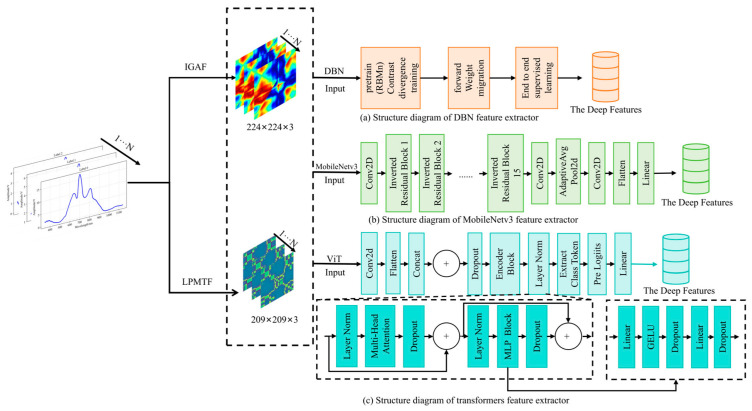
Structure diagram of 2D deep feature extractor.

In order to obtain deep features from feature images, three feature extraction models, DBN, MobileNetv3 and ViT, were developed to process two types of encoded images of Vis/NIR spectral signals from different degrees of moldy pear core. The construction methodology for these deep-level feature extractors is presented in Figure 7.

DBN functions as a generative deep learning model that constructs multi-layered architectures through stacked Restricted Boltzmann Machines (RBMs). The network employs a two-phase learning approach combining unsupervised pre-training with supervised fine-tuning, enabling it to discover inherent distributional patterns within data and demonstrate robust feature learning capabilities when processing high-dimensional feature spaces. The core mechanism of DBNs lies in learning probabilistic distribution features through the energy function of RBMs. This energy function is defined as Formula (4).

Based on this energy function, the conditional probability distributions between hidden and visible layers are derived as Formulas (5) and (6), where $\frac{1}{1+e^{-x}}$ represents the sigmoid activation function, $a_{i}$ and $b_{i}$ are bias terms, and $w_{\mathrm{ij}}$ denotes the connection weights between layers. Through this layer-wise greedy training methodology, DBNs successfully derive abstract feature representations from input data by optimizing these probabilistic relationships across the network hierarchy.

(4) $$E(v,h)=-\sum_{i}a_{i}v_{i}-\sum_{j}b_{j}h_{j}-\sum_{i,j}v_{i}h_{j}w_{ij}$$

(5) $$P\left(h_{j}=1|v\right)=\sigma \left(b_{j}+\sum_{i}v_{i}w_{ij}\right)$$

(6) $$P\left(v_{i}=1|h\right)=\sigma \left(a_{i}+\sum_{j}h_{j}w_{ij}\right)$$

MobileNetv3 implements an inverted residual structure combined with depthwise separable convolutions, achieving efficient feature extraction through its lightweight architectural design. The network incorporates Squeeze-and-Excitation (SE) attention modules and HardSwish activation functions, which enhance local feature extraction capabilities while maintaining computational efficiency. The core efficiency mechanism of MobileNetv3 lies in its depthwise separable convolution approach, which decomposes standard convolution operations into depthwise and pointwise components. This decomposition substantially reduces both parameter count and computational overhead. The computational complexity of standard convolution is expressed as Formula (7). In contrast, the computational complexity of depthwise separable convolution is expressed in Formula (8). This yields a computational complexity reduction ratio shown in Formula (9), where $D_{K}$ represents the kernel size, *M* and *N* denote the input and output channel numbers, respectively, and $D_{F}$ indicates the feature map size. Through this mathematical optimization and architectural innovation, MobileNetv3 successfully improves the efficiency of deep feature extraction while maintaining robust performance across various computer vision tasks.

(7) $${\mathrm{Cost}}_{Standard}=D_{K}\times D_{K}\times M\times N\times D_{F}\times D_{F}$$

(8) $${\mathrm{Cost}}_{DSC}=D_{K}\times D_{K}\times M\times D_{F}\times D_{F}+M\times N\times D_{F}\times D_{F}$$

(9) $$\frac{Cost_{DSC}}{Cost_{Standard}}=\frac{1}{N}+\frac{1}{D_{K}^{2}}$$

ViT employs self-attention mechanisms and the Transformer framework to extract global features from images. This network partitions input images into fixed-size patches, establishing long-range dependencies between patches through positional encoding and multi-head self-attention mechanisms, which effectively capture global feature representations of images. The core of ViT lies in the multi-head self-attention mechanism, which captures global feature dependencies by computing attention weights between image patches. The self-attention mechanism is calculated as Formula (10), where *Q*, *K*, and *V* represent the query, key, and value matrices, and $d_{k}$ is the dimension of the key vector. The multi-head attention mechanism, as described in Formula (11), enhances feature representation capability by computing multiple attention heads in parallel.

(10) $$Attention(Q,K,V)=softmax\left(\frac{QK^{T}}{\sqrt{d_{k}}}\right)V$$

(11) $$MultiHead(Q,K,V)=Concat({\mathrm{head}}_{1},\ldots ,{\mathrm{head}}_{h})W^{O}$$

This mechanism enables ViT to establish long-range dependencies between image patches, effectively capturing global spatial feature patterns of pear core internal structures. Compared with conventional convolutional neural networks, ViT exhibits superior global modeling capabilities when processing complex textural features, making it particularly well-suited for tasks requiring a comprehensive understanding of spatial relationships across the entire image. This ViT model uses a 16 × 16 patch size, 12 Transformer layers, and 12 multi-head attention heads, following the standard ViT-Base architecture.

### 2.7. Development of a Classification Model for Moldy Pear Cores

SVM employing radial basis function kernels constitutes a supervised learning paradigm exhibiting high efficacy for multi-category classification problems. SVM works by finding optimal hyperplanes that maximally separate different classes in the feature space through the margin maximization principle. The core idea of SVM is to construct decision boundaries that can effectively distinguish between different categories while maintaining good generalization capability [29]. The fundamental mechanism of SVM involves projecting training data into a higher-dimensional feature space, wherein distinct classes are discriminated by establishing optimal decision boundaries. The corresponding basic principle is presented in Formula (12), where f(x) represents the output of the classification function, $x_{i}$ are the Lagrange multipliers, $y_{i}$ are the class labels. $K(x_{i},x)$ represents the kernel function that nonlinearly maps the input feature vector x to a high-dimensional space, and b is the bias term. For multi-class classification problems with three categories, SVM employs the One-vs-One (OvO) strategy by default in the scikit-learn implementation. The final prediction is determined by majority voting among binary classifiers, as shown in Formula (13). The optimization objective of SVM is formulated as a quadratic programming problem, as shown in Formula (14), where *C* is the regularization parameter that controls the trade-off between minimizing the classification error and maximizing the margin, and $\xi_{i}$ represents the slack variables that allow some misclassification. In this implementation, the Gaussian RBF kernel function was adopted with kernel coefficient gamma set to ‘scale’, the regularization parameter C was set to 1.0, and probability estimation was enabled to provide prediction confidence scores.

(12) $$f(x)=sign\left(\sum_{i=1}^{n}\alpha_{i}y_{i}K(x_{i},x)+b\right)$$

(13) y=argmaxc∈Y∑1≤i<j≤3II(fi,(x)j=c)

(14) $$min_{w,b,\xi }\frac{1}{2}||w|{|}^{2}+C\sum_{i=1}^{n}\xi_{i}$$

### 2.8. Model Performance Assessment

Model performance against the testing data was quantified via accuracy, precision, recall, and $F_{1}$-score. Their formulas and definitions are shown in Formulas (15)–(18). All metrics were calculated as macro-averaged values to account for class imbalance. Statistical significance between classifiers was assessed using McNemar’s test. Confusion matrices were employed to evaluate per-class classification performance.

(15) $$Accuracy=\frac{TP+TN}{TP+TN+FP+FN}$$

(16) $$Precision=\frac{TP}{TP+FP}$$

(17) $$Recall=\frac{TP}{TP+FN}$$

(18) $$F_{1}-Score=\frac{2\times Precision\times Recall}{Precision+Recall}$$

To alleviate sampling bias, the dataset was divided into training and test sets via stratified random sampling, ensuring consistent class distribution across both subsets. The training set comprised approximately 85%, totaling 1052 examples, with 540 healthy pear samples, 186 slightly moldy pear core samples, and 326 pear samples with severely moldy pear core. Furthermore, 20% of the training data were randomly sampled and allocated for validation subset for hyperparameter optimization and model selection. The test set comprised approximately 15%, totaling 196 examples, with 102 healthy pear samples, 32 slightly moldy pear core samples, and 62 pear samples with severely moldy pear core. All performance indicators were calculated based on the premise that testing data remained statistically independent of model training and originated from the same population distribution. This prerequisite was strictly maintained across all experimental protocols.

## 3. Results and Discussion

### 3.1. Comparative Analysis of Pear Feature Images

**Figure 8:**
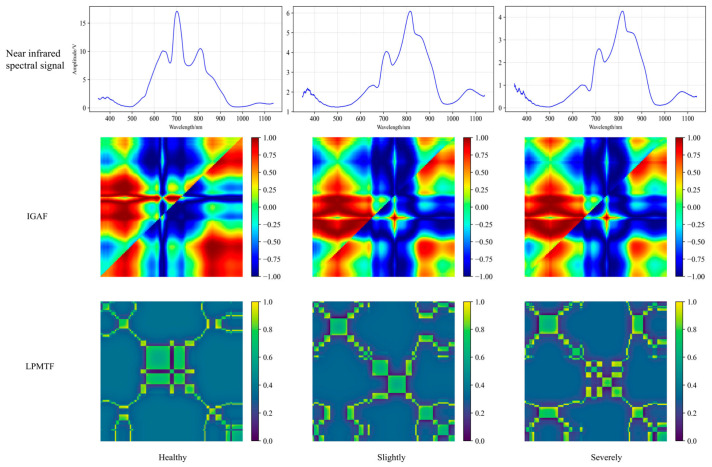
Vis/NIR spectral signals and feature images of pears with different degrees of mold. Note: The three columns (left to right) represent healthy, slightly moldy, and severely moldy pear samples, respectively. The first row shows the raw Vis/NIR spectral signals. The second row presents the IGAF-encoded images, which exhibit similar visual patterns across categories. The third row displays the LPMTF-encoded images, which show distinct textural variations among the three severity levels, providing clearer visual discrimination for classification.

As shown in Figure 8, although spectral curves exhibit subtle variations across different severity levels of moldy pear core, these differences are visually inconspicuous and insufficient for direct interpretation or accurate classification. The spectral absorption peaks are primarily concentrated within the 600–900 nm range, with their amplitudes decreasing progressively as disease severity increases. This trend arises from fundamental changes in light–tissue interactions: healthy pear tissues contain air-filled intercellular spaces where light attenuation is dominated by scattering, whereas moldy pear core triggers enzymatic browning in the core and adjacent flesh tissue. Elevated polyphenol oxidase activity drives oxidation of the core and adjacent flesh, producing melanin-like pigments that enhance light absorption while reducing transmittance, consequently lowering the spectral energy values detected.

Given these complex, overlapping spectral signatures, relying solely on raw spectral curves proves inadequate for precise discrimination among disease severity levels. The IGAF-encoded images proved insufficient for robust generalization, particularly in distinguishing between slight and severe infections on independent test data. LPMTF representations revealed distinct textural patterns across all three severity categories. These pronounced textural variations provide the critical discriminative information necessary for accurately identifying and grading moldy pear core.

### 3.2. Performance of Characteristic Wavelength Extraction Models

**Figure 9:**
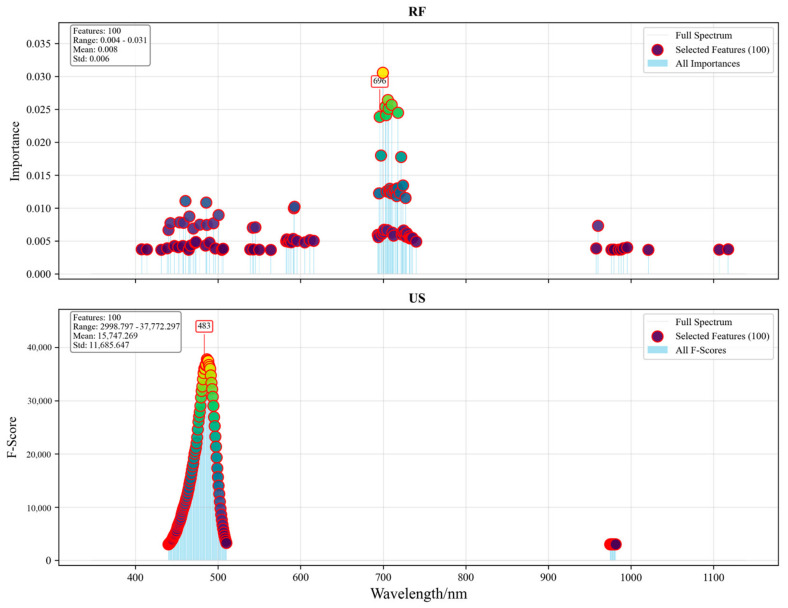
Distribution diagram of selected features.

These two feature selection methods identified the characteristic wavelengths from the original Vis/NIR spectral data. Figure 9 illustrates the distribution of selected characteristic wavelengths for each method over the entire spectral range. Both methods selected 100 wavelength features. The features selected by the random forest importance assessment method were widely distributed, mainly in the vicinity of 400–600 nm, around 700 nm, and near 1000 nm. The most important wavelength identified was 696 nm, related to chlorophyll. The features selected by the univariate selection method were all concentrated near 500 nm. The most important wavelength identified was 483 nm, corresponding to chlorophyll absorption bands [30]. The extracted characteristic wavelengths were subsequently employed as input features for the SVM model. We then evaluated their discriminative capability for distinguishing between different moldy pear core degrees.

The random forest importance assessment method achieved a higher classification performance with 93.43% accuracy, demonstrating a substantial improvement of approximately 10 percentage points over the univariate selection method (83.33% accuracy). The RF method also exhibited excellent precision (94.37%) and $F_{1}$-score (94.01%), suggesting consistent performance across all metrics. The comprehensive performance comparison is presented in Table 1.

### 3.3. Training and Optimization of Neural Network Models

#### 3.3.1. 1D-ResNet Model Training and Optimization

The 1D-ResNet model was specifically designed for processing sequence characteristics of Vis/NIR spectral data. The 1D-ResNet framework comprises multiple residual modules incorporating skip connections, thereby mitigating the vanishing gradient phenomenon prevalent in deep architectures. The key parameters and their optimized values are presented in Table 2.

**Figure 10:**
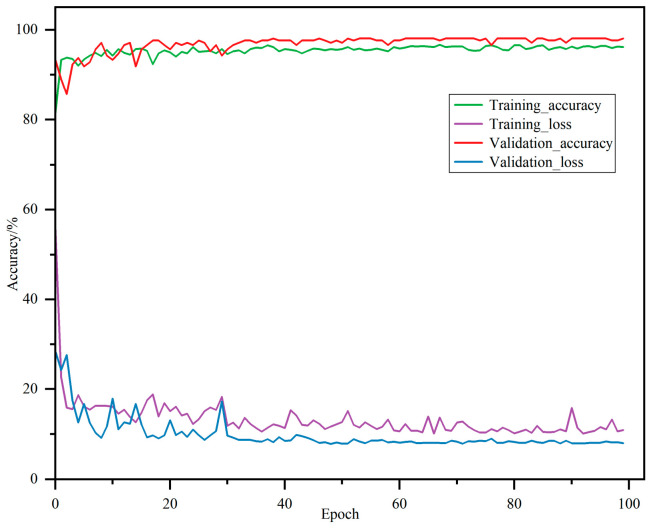
Training and validation curves of 1D-ResNet model.

The training curves of the 1D-ResNet model are shown in Figure 10. The model achieved stable convergence in the 100th epoch, with a training accuracy of 95.83% and a validation accuracy of 98.09%. The learning curve showed that the training and validation performance were improving simultaneously. During 100 epochs of training, the training loss gradually decreased from 0.56 to 0.11; the validation loss also decreased from 0.28 to 0.08, which meant that the model attained the best performance equilibrium in the later stages of training. However, despite the good performance on the validation set, the model’s generalization capability on the independent test set required further evaluation.

#### 3.3.2. 2D Deep Learning Model Training Results

**Figure 11:**
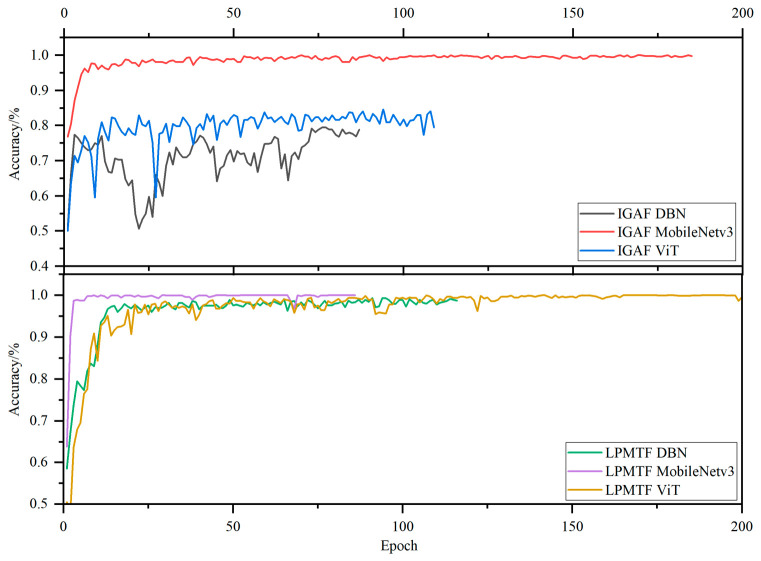
Training accuracy process of 2D deep learning model.

Deep Learning models require the conversion of 1D spectral data into a 2D representation to leverage the spatial feature extraction capabilities of these architectures. Key parameters and their optimized values are shown in Table 3. Early stopping was enabled during the training of all models to save training time. To prevent premature stopping and insufficient model training, the early stopping patience was set to 50 epochs. Images converted using the IGAF method were trained for 87, 185, and 109 epochs in the DBN, MobileNetv3, and ViT models respectively. Images converted using the LPMTF method were trained for 117, 86, and 244 epochs in the DBN, MobileNetv3, and ViT models, respectively. The training processes of the 2D models is shown in Figure 11. The training curves revealed that for IGAF-encoded data, only the MobileNetv3 model converged to 99.68% accuracy, while DBN and ViT only reached around 80%, with significant fluctuations in accuracy. Under LPMTF encoding, all three models achieved significantly higher training accuracy compared to IGAF encoding. Among them, the ViT model achieved a training set accuracy close to 100%, and exhibited high stability and low fluctuations after convergence, indicating superior convergence behavior for the LPMTF-ViT combination.

### 3.4. Comparative Performance Evaluation of Different Detection Methods

**Figure 12:**
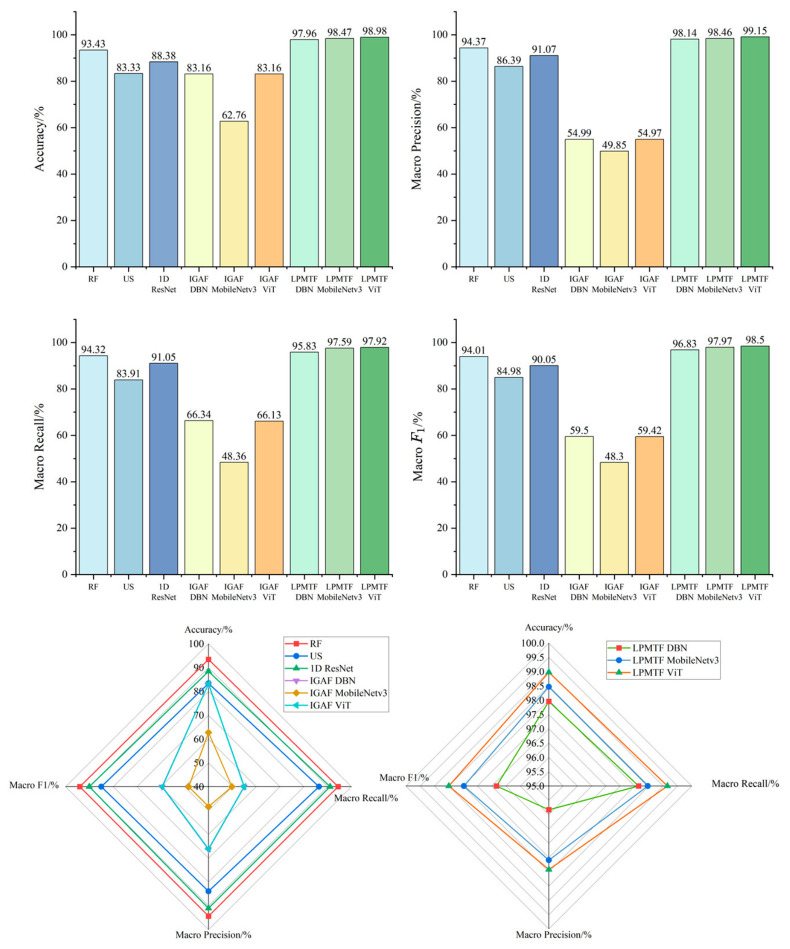
Performance indicators of all methods.

Performance evaluation metrics for different classifiers are depicted as bar charts and radar plots in Figure 12. Statistical significance was assessed using McNemar’s test on the test set (n = 196). LPMTF-ViT markedly exceeded the traditional RF-SVM method (98.98% vs. 93.43%, χ^2^ = 28.03, *p* < 0.001).

Among traditional machine learning methods, RF attained superior classification performance to US (93.43% vs. 83.33%), attributable to its wider wavelength distribution and ensemble-based feature selection. However, the 1D-ResNet exhibited poor generalization, with test accuracy (88.38%) substantially lower than training accuracy (95.83%), indicating overfitting to the high-dimensional (1044-dimensional) spectral data.

The deep learning models results revealed critical encoding-dependent performance gaps. Under identical ViT architecture, LPMTF encoding significantly outperformed IGAF encoding (98.98% vs. 83.16%, χ^2^ = 6.75, *p* = 0.009), demonstrating that the multi-scale Markov transition representation captured more discriminative spectral dynamics than the Gramian angular field. Notably, MobileNetv3 trained on IGAF features showed severe overfitting (99.68% training vs. 62.76% test accuracy), suggesting that IGAF captures spurious patterns rather than discriminative patterns. This stemmed from GAF’s inherent limitations: its polar coordinate transformation ($\varphi =\arccos({\tilde{x}}_{i}))$ encodes static angular relationships while remaining invariant to amplitude variations, thereby losing critical spectral magnitude information [31]. In contrast, MTF captures dynamic transition probabilities across quantile bins, encoding the temporal evolution of spectral features. Consequently, all three architectures (DBN, MobileNetv3, ViT) achieved high accuracy when using LPMTF encoding, with ViT attaining optimal performance (98.98% accuracy, 99.15% macro precision, 97.92% macro recall, and 98.50% $F_{1}$-score).

These results suggest that transforming 1D spectral signals into 2D images can unlock the potential of powerful computer vision models, and the choice of both the encoding method and the network architecture is critical to achieving optimal performance.

### 3.5. Ablation Analysis of Moldy Pear Core Classification Model

This study presents a model that integrates the ViT architecture with the LPMTF transformation method. To assess its feasibility and effectiveness, we conducted two types of ablation studies using the same training and test set partitions as described in Section 2.8: (i) LPMTF component removal, and (ii) ViT attention mechanism elimination. The experimental outcomes are summarized in Table 4. The baseline model employed a parameter configuration of equal-frequency quantile interval q = 12 and piecewise aggregate approximation window size window_size = 5. When adjusting either the q value or window_size, the model’s performance metrics exhibited various degrees of decline, indicating that parameter selection significantly influences classification performance, and the parameter combination adopted in this research proved optimal. Notably, the accuracy dropped from 98.98% to 52.04% because ViT’s attention mechanism is essential for capturing the long-range dependencies within LPMTF feature maps. This encoding transforms time-series into images where class-discriminative patterns rely on relationships between distant quantile bins and time windows. Removing the attention mechanism eliminated the network’s capacity to capture these global spectral correlations, reducing it to a patch-wise MLP that processes local regions in isolation. Consequently, the model could not decode the global topological structure embedded in the LPMTF representations, resulting in a substantially degraded performance. These ablation studies validate the synergistic effectiveness of combining LPMTF with ViT and underscore the necessity of parameter optimization.

### 3.6. Comparative Analysis of DBN, MobileNetv3, and ViT Architectures

The performance differences among the three architectures can be attributed to their fundamentally distinct feature extraction mechanisms. DBN, as a generative model based on stacked Restricted Boltzmann Machines, learns hierarchical probabilistic representations through a two-phase strategy combining layer-wise unsupervised pre-training with supervised fine-tuning. However, its fully connected structure processes flattened input vectors, thereby losing the spatial topology of 2D images and limiting its ability to capture spatial dependencies within encoded spectral representations. MobileNetv3 employs depthwise separable convolutions combined with Squeeze-and-Excitation (SE) attention modules, enabling efficient local feature extraction with substantially reduced computational complexity. While this lightweight architecture effectively captures local spatial patterns, its inherently limited receptive field constrains the modeling of long-range dependencies across the entire image. ViT, through its multi-head self-attention mechanism, establishes direct relationships between all image patches simultaneously, enabling comprehensive global feature modeling without the need for progressive receptive field expansion. This capability is particularly critical for interpreting LPMTF-encoded images, where class-discriminative patterns depend on the transition relationships between different quantile states and the contextual information across multi-scale temporal windows. The ablation study in Section 3.5 further confirmed this point: removing ViT’s attention mechanism resulted in a dramatic accuracy decline from 98.98% to 52.04%, indicating that global attention is the key mechanism for effectively decoding LPMTF representations. Notably, the performance differences among architectures were observed under LPMTF encoding; when using the less informative IGAF encoding, all three architectures failed to achieve satisfactory performance, suggesting that the choice of encoding method serves as a prerequisite for achieving high detection accuracy. These findings highlight the critical role of ViT’s architecture in achieving superior detection performance.

The superior performance of ViT can be fundamentally attributed to its distinctive architectural design that treats an image as a sequence of fixed-size patches. In the context of this study, each IGAF-encoded image is partitioned into 16 × 16 non-overlapping patches, yielding a sequence of 196 patch tokens, while each LPMTF-encoded image yields a sequence of 169 patch tokens. Each patch is linearly projected into an embedding vector and augmented with positional encoding to preserve spatial ordering. This sequence-based representation offers three key structural advantages over convolutional architectures. First, the self-attention mechanism computes pairwise attention weights between all patches in a single operation, establishing direct connections between any two regions of the image regardless of their spatial distance. This eliminates the locality constraint inherent in convolutional networks, where information can only flow between distant regions through successive layers of small receptive fields. Second, the multi-head attention mechanism enables the model to simultaneously attend to different types of relationships across the image—such as correlations between quantile state transitions at different temporal scales in the LPMTF representation—through parallel attention heads, each learning distinct dependency patterns. Third, the Transformer architecture maintains uniform computational pathways across all layers, avoiding the information bottleneck caused by progressive spatial downsampling in convolutional networks, which can dilute fine-grained spectral features encoded in the LPMTF images. These structural properties make ViT particularly well-suited for analyzing LPMTF representations, where the discriminative information is encoded in the global topological relationships among quantile state transitions rather than in isolated local patterns. In summary, ViT achieved the best performance owing to its superior global spatial modeling capability, while MobileNetv3 provided a favorable balance between accuracy and computational efficiency, and DBN was constrained by its inability to preserve spatial structural information.

### 3.7. External Validation

To verify the ability of the LPMTF combined with ViT to classify moldy pears, this study conducted an external experiment using an independent test set. A total of 72 cases were randomly selected from newly purchased samples, including 30 healthy pears, 19 slightly moldy pears, and 23 severely moldy pears, forming an imbalanced independent validation set. A confusion matrix was used to evaluate and analyze the classification ability of the model.

**Figure 13:**
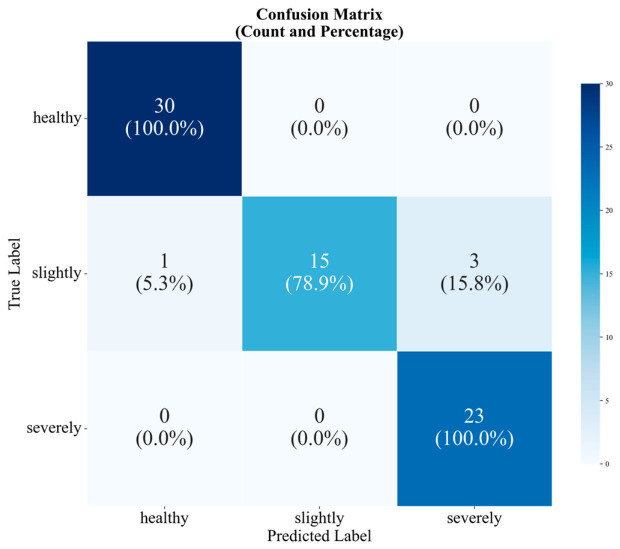
The confusion matrix of LPMTF combined with ViT.

As shown in Figure 13, the confusion matrix of LPMTF combined with ViT showed the accuracy of the classification model for healthy, slightly, and severely moldy pear core samples was 100%, 78.9%, and 100%, respectively, with an overall accuracy of 94.44%. Notably, the reduced accuracy for slightly moldy samples stemmed from the limited infection area in early disease stages, where weak spectral perturbations are easily confounded by inherent biological variability among individual pears. This indicates that the LPMTF-ViT model has good discriminative ability for moldy pear, though early-stage detection remains challenging due to natural sample heterogeneity.

For the independent test dataset, the classification efficacy of the trained LPMTF combined with the ViT model was benchmarked against prior literature. As presented in Table 5, both acoustic vibration signals and Vis/NIR spectral signals achieved good results in detecting apple mold cores, demonstrating high detection accuracy. The Vis/NIR spectroscopy method obtained the highest accuracy of 98.75% in apple mold core detection [32], while the acoustic vibration signal method reached the highest accuracy of 96.97%. However, acoustic vibration signals are highly sensitive to environmental vibrations, equipment mechanical noise, console shaking, and external sound waves. Fruit size, shape, stem calyx direction, and skin thickness can all change the overall stiffness and modal distribution. In contrast, Vis/NIR spectroscopy has higher robustness to geometric changes and lower requirements for external factors. Zhang et al. [33] and Li et al. [34] employed acoustic sensors to identify internal disorders in pear fruit, with an overall accuracy of 93.90% and 93.66%. In comparison, the LPMTF combined with the ViT model used in this study had a higher overall accuracy. It is noteworthy that discrepancies in sample composition and experimental protocols across studies may account for variations in reported classification accuracy.

To further validate the transferability of the LPMTF-ViT model, subsequent studies should move beyond the detection of moldy pear core in Korla pears from a single origin. It is imperative to assess its versatility regarding other pear cultivars and even distinct fruit species. The framework manifested superior discriminative capacity for categories exhibiting fine-grained internal distinctions under stable production conditions. Although the specimens in this study were all obtained from a single origin with consistent collection conditions, the LPMTF-ViT architecture has already displayed excellent and stable classification performance when trained with appropriately varied data. This implies its capability for effective utilization across an expanded scope of internal defect detection applications. Future research should include experiments on datasets from multiple production regions, cultivars, and under fluctuating growth, harvesting, and storage conditions. This will be vital to further confirm and strengthen the model’s practical utility and reliability for field-based agricultural and supply chain operations.

## 4. Conclusions

This study developed a comprehensive comparative framework for the nondestructive detection of moldy pear core using Vis/NIR spectroscopy combined with advanced image encoding methods and deep learning techniques. The research systematically evaluated three detection strategies: traditional machine learning with characteristic wavelength extraction, 1D-CNN, and deep learning model with improved encoding transformations.

The results demonstrated that the LPMTF combined with ViT achieved superior performance with an overall classification accuracy of 98.98% on the test set, significantly outperforming traditional methods (RF: 93.43%, US: 83.33%) and 1D-ResNet (88.38%). The LPMTF-ViT model exhibited excellent metrics with macro Precision of 99.15%, macro Recall of 97.92%, and macro $F_{1}$-score of 98.50%. Independent validation confirmed the model’s effectiveness with an overall accuracy of 94.44%, demonstrating competitive performance compared to existing literature.

This investigation offers non-invasive detection methodologies for early diagnosis of internal disorders in fruit commodities, alongside methodological support for automated quality assurance in the fruit industry. The decreasing cost of Vis/NIR equipment and advances in edge computing make this method potentially deployable on industrial sorting lines, thus practically accessible to producers.

Nevertheless, several limitations warrant acknowledgment. First, independent external experiments showed that the model has significant limitations in the detection of slightly moldy pear core. Further research is needed to improve the accuracy of detection. Secondly, all the experimental samples were collected from a single geographical area and limited to one pear variety, which limited the universality of the research results to other climate and soil conditions. Spectral data were obtained only under controlled laboratory conditions without considering potential interference in field settings. To improve extrapolation, future studies should expand sample diversity across regions and cultivars, and explore domain adaptation techniques. Furthermore, integrating this method with complementary approaches, including destructive laboratory analyses (e.g., microbial culture, mycotoxin quantification) and sensory evaluation, would strengthen practical reliability through multi-modal validation. Future research should expand the diversity of samples from multiple sources and varieties to enhance the practical utility and widespread adoption of the proposed method in agricultural applications.

## Figures and Tables

**Table 1:** Comparison of Specific Performance of Feature Wavelength Extraction.

	M-Accuracy (%)	M-Precision (%)	M-Recall (%)	M-$F_{1}$ (%)
RF	93.43	94.37	94.32	94.01
US	83.33	86.39	83.91	84.98

Note: M- indicates macro-average.

**Table 2:** Parameter Table of 1D-ResNet Model.

Input Dimension	1044
Batch size	64
Learning rate	0.01
Weight decay	0.0001
Epochs	100
Dropout rate	0.3
Optimizer	Adam

**Table 3:** Parameter Table of 2D deep learning models.

Model	MobileNetv3	ViT	DBN
Batch size	64	32	16
Learning rate	CosineAnnealingLR	CosineAnnealingLR	CosineAnnealingLR
Weight decay	0.0001	0.02	0.0001
Dropout rate	0.2	0.1	0.3
Optimizer	Adam	AdamW	Adam
Max epochs	250	250	250
Patience	50	50	50

**Table 4:** Ablation Experiment Results.

LPMTF Parameters		Attention Mechanism	M-Accuracy/%	M-Precision/%	M-Recall/%	M-$F_{1}$/%
q	window_size					
12	10	Enabled	98.47	98.83	96.88	97.77
6	5	Enabled	95.92	96.46	94.82	95.54
12	5	Disabled	52.04	17.35	33.33	22.82
12	5	Enabled	98.98	99.15	97.92	98.50

Note: M- indicates macro-average.

**Table 5:** Comparative analysis of classification performance against existing literature.

Research Objects	Detection Method	Overall Accuracy (%)	References
Apple	NIR	98.75	[32]
Apple	NIR	94.44	[35]
Apple	Acoustic vibration	96.97	[22]
Apple	Acoustic vibration	96.70	[36]
Pear	Low radio frequency capacitive	89.50	[37]
Pear	Acoustic vibration	93.90	[33]
Pear	Acoustic vibration	93.66	[34]
Pear	NIR	94.44	This paper

## Data Availability

The original contributions presented in this study are included in the article. Further inquiries can be directed to the corresponding author.

## Abbreviations

The following abbreviations are used in this manuscript:

Vis/NIR	Visible-near-infrared
1D-CNN	One-dimensional convolution neural network
GAF	Gramian angular field
IGAF	Improved gramian angular field
GADF	Gramian angular difference field
GASF	Gramian angular sum field
LP	Laplacian pyramid
LPMTF	Laplacian pyramid Markov transition field
MTF	Markov transition field
SVM	Support vector machine
ViT	Vision Transformer
DBN	Deep Belief Network
US	Univariate selection
RF	Random forest
